# Scalable Synthesis of Freestanding Sandwich-structured Graphene/Polyaniline/Graphene Nanocomposite Paper for Flexible All-Solid-State Supercapacitor

**DOI:** 10.1038/srep09359

**Published:** 2015-03-23

**Authors:** Fei Xiao, Shengxiong Yang, Zheye Zhang, Hongfang Liu, Junwu Xiao, Lian Wan, Jun Luo, Shuai Wang, Yunqi Liu

**Affiliations:** 1 Laboratory for Large-Format Battery Materials and System, Ministry of Education Advanced Optoelectronic/Energy Materials and Interface Chemistry Joint Laboratory School of Chemistry & Chemical Engineering Huazhong University of Science and Technology Wuhan 430074, P. R. China; 2 National Center for Electron Microscopy, School of Materials Science and Engineering, The State Key Laboratory of New Ceramics and Fine Processing, Key Laboratory of Advanced Materials (MOE), Tsinghua University, Beijing 100084, P. R. China; 3 Beijing National Laboratory for Molecular Sciences, Institute of Chemistry, Chinese Academy of Sciences Beijing 100190, P. R. China

## Abstract

We reported a scalable and modular method to prepare a new type of sandwich-structured graphene-based nanohybrid paper and explore its practical application as high-performance electrode in flexible supercapacitor. The freestanding and flexible graphene paper was firstly fabricated by highly reproducible printing technique and bubbling delamination method, by which the area and thickness of the graphene paper can be freely adjusted in a wide range. The as-prepared graphene paper possesses a collection of unique properties of highly electrical conductivity (340 S cm^−1^), light weight (1 mg cm^−2^) and excellent mechanical properties. In order to improve its supercapacitive properties, we have prepared a unique sandwich-structured graphene/polyaniline/graphene paper by in situ electropolymerization of porous polyaniline nanomaterials on graphene paper, followed by wrapping an ultrathin graphene layer on its surface. This unique design strategy not only circumvents the low energy storage capacity resulting from the double-layer capacitor of graphene paper, but also enhances the rate performance and cycling stability of porous polyaniline. The as-obtained all-solid-state symmetric supercapacitor exhibits high energy density, high power density, excellent cycling stability and exceptional mechanical flexibility, demonstrative of its extensive potential applications for flexible energy-related devices and wearable electronics.

Ultrathin and flexible energy storage devices are of considerable current interest because of the increasing demands for modern electronic such as soft electronic equipment, roll-up displays, stretchable integrated circuits, and portable medical products^1^,^2^,^3^,^4^. Supercapacitors (SCs), also known as electrochemical capacitors or ultracapacitors, are promising energy storage devices that bridge the gap between batteries and conventional capacitors. SCs can provide high specific power (10 kW kg^−1^), long cycle life (3 × 10^5^ cycles), and fast charge/discharge processes (within seconds), and therefore stimulate extensive interests as an alternative or supplement to batteries in the energy storage field^5^,^6^,^7^,^8^. The design of flexible SCs demands flexible electrodes with favorable mechanical strength and large capacitance. For the development of flexible electrodes in SCs application, two typical strategies are adopted. One is to load active materials on flexible substrates such as carbon fabrics^9^, cellulose^10^, nanoporous gold^11^ and nickel foam^12^,^13^. The other is to fabricate freestanding and soft electrodes. Papers^14^, films^15^ and clothes^16^ made from carbon materials have been demonstrated to be suitable for this purpose. In comparison with the former method, the freestanding electrodes can significantly simplify cell packing by eliminating inactive ingredients such as binders and current collectors, and hence improve the overall performance when the total mass of the device is taken into account, which holds great promising for the development of ultrathin, lightweight and high-performance SCs.

Carbon-based nanomaterials have attracted enormous scientific and technological interest for their potential applications in energy storage and conversion such as ultracapacitors, batteries, fuel cells and solar cells. Among the large family of carbon materials, graphene, consisting of a single-layer of sp^2^-hybridized carbon atoms, has been paid the most intensive attention because of its excellent electrical conductivity, superior mechanical flexibility, high theoretical surface area and relatively inert electrochemistry^17^,^18^,^19^. More importantly, recent reports by several groups including us have shown that graphene nanosheets can be assembled into freestanding and flexible 3D graphene foam^20^, or paper-like structure via vacuum filtering^21^,^22^, self-assembly^23^,^24^,^25^, pressing graphene aerogel^26^ or hydrogel^27^ and drying on petri dish^28^. However, for the preparation of graphene paper, these methods either have complicated procedure of manufacturing or cannot produce graphene paper with large area, which greatly limit its large-scale production. On the other hand, the double-layer capacitance originated form pristine graphene material is much lower than the pseudocapacitance of conductive polymers or transition metal oxides. Therefore, it is still a great challenge to develop a novel and facile method to fabricate freestanding and flexible graphene paper with high conductivity, good capacitive performance and favorable mechanical strength that can be scaled up for widespread commercialization.

In this work, we present a facile method to prepare a new type of sandwich-structured graphene/polyaniline/graphene nanohybrid paper with desirable properties of high conductivity, good chemical stability and excellent mechanical robustness. Large-scale graphene paper used in this study was fabricated by a novel printing technique and subsequent CO_2_ bubbling delamination method. In order to increase the energy storage capacity of graphene paper, polyaniline (PANI) nanofibers were in situ electropolymerized on it^29^. Utilizing both Faradaic and non-Faradaic processes to store charge, the PANI nanofibers modified graphene (graphene/PANI) hybrid paper electrode could achieve high energy and power densities without sacrificing the cycling stability and affordability. More importantly, our results have showed that the following dip coating of an ultrathin graphene layer on the surface of the porous PANI nanofibers leads to the formation of a graphene/PANI/graphene nanohybrid paper with a unique sandwich structure, which further increases the specific capacitance and improves the cycling stability of the paper electrodes. The freestanding graphene/PANI/graphene nanocomposite paper was used as binder-free electrode to fabricate all-solid-state symmetric SC with polymer gel as electrolyte. The resultant flexible and light-weight SC exhibited high energy density, high power density and excellent cycling stability. Particularly, its volumetric energy density outperforms many previously reported solid-state SCs, such as polypyrrole-coated paper symmetric SC^10^, carbon/MnO_2_ fiber symmetric SC^30^, H-TiO_2_@MnO_2_//H-TiO_2_@C asymmetric SC^31^ and WO_3-x_/MoO_3-x_ asymmetric SC^32^. It is worth noting that the proposed modular approach for the fabrication of freestanding sandwich-structured graphene nanohybrid paper is not just restricted to the graphene/polyaniline/graphene system, but also contributes to the design of hybrid nanoarchitectures with tailored compositions and functions. These substantially demonstrate the extensive potential applications the proposed graphene nanohybrid paper for flexible energy-related device and wearable electronics.

## Results

**Figure 1:**
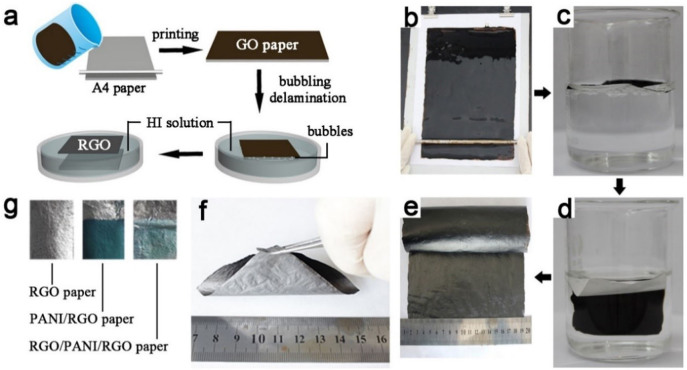
(a) Schematic image illustrating the formation of a freestanding RGO paper. (b) Fabrication process of GO film by the printing method. (c, d) Peeling GO film from the printing paper with the bubbling delamination method in 1 M HCl (since HI is dark colored, we used HCl instead to illustrate this process). (e) Photograph of large-scale RGO paper. (f) Photograph of RGO paper with excellent flexibility. (g) Photograph of RGO, PANI/RGO and RGO/PANI/RGO paper.

Fig. 1a schematically describes the procedure for preparing graphene-based nanohybrid paper. The graphene oxide (GO) paper was firstly fabricated by spreading GO solution on a piece of standard commercial A4 paper via a facile and scalable printing technique (Fig. 1b). The area of GO paper can be freely adjusted by choosing different sized cellulose paper substrate, which provides the possibility to produce large-area graphene paper. Moreover, the thickness of GO paper can also be tailored in a wide range by using different volume and concentration of GO dispersion. Aftrer that, the resultant GO paper was chemically reduced using hydroiodic acid (HI) solution and simultaneously peeled off from A4 paper via a bubbling delamination method to form a freestanding reduced GO (RGO) paper. As demonstrated in Fig. 1c and 1d, when the A4 paper coated GO film was soaked in acids such as HCl, H_2_SO_4_ and CH_3_COOH, a lot of bubbles generated and provided a gentle force to delaminate GO film from the commercial A4 paper. These bubbles produced from the chemical reaction between the additions of the A4 paper and HCl are identified to be CO_2_ bubbles (Fig. S1). If the acid has reducing property, GO paper would be peeled off and reduced simultaneously. During the bubbling delaminating process, the CO_2_ bubbles generated from the cellulose paper substrate prompt the graphene film peeling off the paper in a self-releasing way, which enable us to produce ultrathin freestanding graphene paper with a thickness of 800 nm (Fig. S2) or thick graphene paper with a thickness more than 100 μm. Electrochemical delamination methods have previously been used to transfer graphene grown on Cu^33^ or Pt^34^ foil by H_2_ bubbles at the interface of the graphene and metal substrates. Compared with these reported method, our strategy to delaminate printed graphene film from paper substrate by CO_2_ bubbles is more facile, effective and scalable. The obtained RGO paper exhibits highly electrical conductivity of 340 S cm^−1^, light weight of 1 mg cm^−2^ and excellent mechanical flexibility, which allows it to be rolled up, twisted or bent to any curvature (Fig. 1e and 1f). Furthermore, the reproducibility of the proposed method for the fabrication of graphene paper has also been evaluated. The electrical conductivity (S cm^−1^) and mass thickness (mg cm^−2^) of ten graphene papers display the relative standard deviation (RSD) less than 5.0% (Fig. S3), confirming that this method is highly reproducible. These features enable it a robust substrate for the further electropolymerization of PANI nanofibers on it. Then, PANI layer with dark green color was uniformly coated on the RGO paper, which was denoted by PANI/RGO. The weight ratio of RGO and PNAI in PANI/RGO paper has been measured to be 3:2. After dip-coating and reduction, the PANI/RGO paper was completely covered by a layer of RGO film, as shown in Fig. 1g, and the sandwich-structured RGO/PANI/RGO paper was obtained. The RGO film that coated on the surface of PANI/RGO is ultrathin, so that its weight is negligible. The weight ratio of RGO and PNAI in RGO/PANI/RGO paper is the same to that in PANI/RGO. The resultant conductive, light-weight and flexible paper-like nanocomposite will facilitate industrial application of SCs, especially the spiral wound SCs.

**Figure 2:**
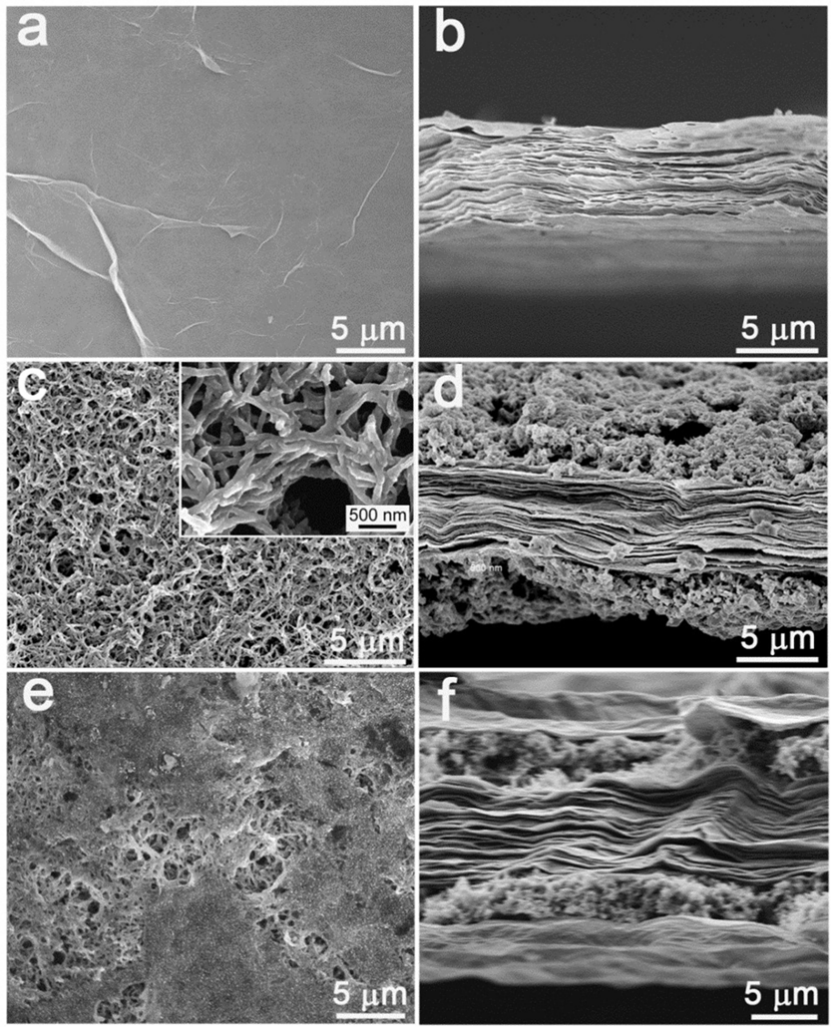
Top view and cross-sectional view SEM images of RGO paper (a, b), PANI/RGO paper (c, d) and RGO/PANI/RGO paper (e, f).

Fig. 2 shows SEM images of different nanohybrid papers. RGO paper exhibits wrinkle-like morphology on its surface (Fig. 2a) and well-layered structure with a uniform thickness of 5 μm throughout its cross-section (Fig. 2b). After in situ electropolymerizing, PANI nanofibers with a diameter of about 70 nm are tangled and twisted to form 3D porous networks on RGO paper (Fig. 2c), which effectively facilitates ions transport between the electrode surface and electrolyte. The SEM image of a cross-section of the PANI/RGO paper reveals that the porous PANI layers are loaded on both sides of RGO paper (Fig. 2d). The as-obtained PANI/RGO paper was then wrapped by an ultrathin RGO layer via dipping in GO solution and reduced by HI. As shown in Fig. 2e, the surface of PANI/RGO paper has been entirely covered by RGO nanosheets, and the PANI nanofibers are sandwiched between RGO layers to form freestanding RGO/PANI/RGO nanohybrid paper (Fig. 2f).

**Figure 3:**
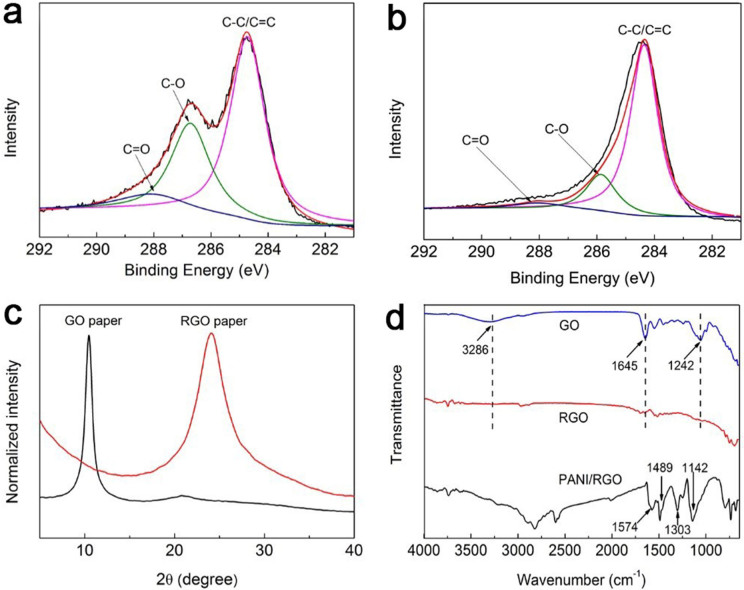
Deconvoluted C1s XPS spectra of (a) GO paper and (b) RGO paper. (c) XRD patterns of GO and RGO papers. (d) FTIR spectra of GO, RGO and PANI/RGO papers.

The deconvoluted C1s XPS spectra of the GO and RGO papers are shown in Fig. 3a and 3b, which suggest that GO paper is highly functionalized with oxygenated groups, such as epoxy/hydroxyl groups (C–O, ~286.5 eV), carbonyl group (C = O, ~288.3 eV) and carboxyl group (O − C = O, ~290.3 eV). After reduced by HI, the C/O ratio increases from 2.5 to 6.6, demonstrative of the elimination of a large fraction of oxygenated groups on GO nanosheets by chemical reduction. Fig. 3c exhibits the XRD patterns of GO and RGO paper. The GO paper exhibits one peak centered at 10.5°, corresponding to the (002) reflection of stacked GO sheets with a layer-to-layer distance (d-spacing) of 8.42 Å. Compared with GO paper, the XRD peak of RGO paper shift to 24.1° with the decrease of d-spacing to 3.69 Å, further confirming the removal of the oxygen-containing groups on GO nanosheets. The FTIR spectrum of the GO paper shows a broad absorption band centered at 2995 ~ 3637 cm^−1^ attributed to O–H stretching vibrations of adsorbed water molecules and structural OH groups, and two absorption band centered at 1645 cm^−1^ and 1242 cm^−1^ ascribed to carboxyl and epoxy stretching vibrations^35^. After being soaked in HI solution for a few minutes, GO paper was then effectively reduced to RGO paper, which was demonstrated by decreased dramatically absorption peak of oxygen functional group. For PANI/RGO paper, the FT-IR spectrum is dominated by that of the PANI (Fig. 3d), the C = C stretching vibrations of quinoid and benzenoid rings, the C-N stretching vibration and C = N stretching vibration appear at 1574, 1489, 1303 and 1142 cm^−1^ respectively, which are in good agreement with previous spectroscopic characterizations of PANI^36^,^37^.

## Discussion

**Figure 4:**
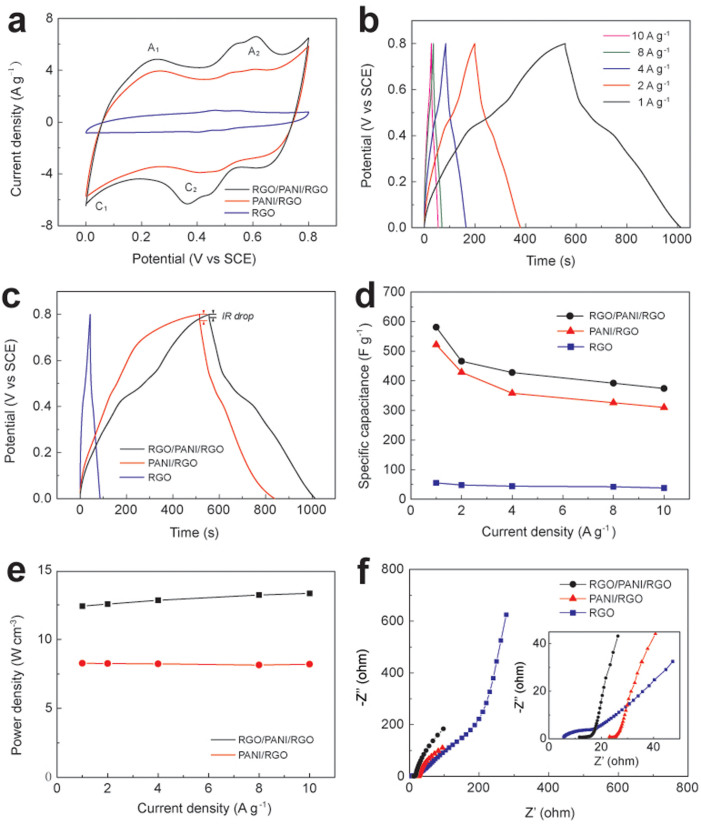
(a) CV curves of RGO/PANI/RGO, PANI/RGO and RGO paper electrodes at a scan rate of 10 mV s^−1^ in 1 M H_2_SO_4_ electrolyte. (b) GV charge/discharge curves of RGO/PANI/RGO paper at different current densities. (c) GV charge/discharge curves of RGO/PANI/RGO, PANI/RGO and RGO paper electrodes at the current density of 1 A g^−1^. (d) Specific capacitances of RGO/PANI/RGO, PANI/RGO and RGO paper electrodes versus discharge current densities. (e) Power densities of RGO/PANI/RGO and PANI/RGO paper electrodes versus discharge current densities. (f) Nyquist plots of RGO/PANI/RGO, PANI/RGO and RGO paper electrodes.

As described above, it will be of great interest to study the electrochemical performance of these nanohybird paper electrodes. The electrochemical measurements were conducted in a three-electrode configuration in 1 M H_2_SO_4_ aqueous solution. As shown in Fig. 4a, the CV curve of RGO paper exhibits a nearly rectangular and symmetric shape, indicative of perfect electrical double-layer capacitance behavior and fast charging/discharging process characteristic. For PANI/RGO and RGO/PANI/RGO papers, two couples of redox peaks (C1/A1, C2/A2) are observed. The pair of redox peaks C1/A1 is due to the redox transitions between the semiconducting-state form (leucoemeraldine form) and a conducting state (polaronic emeraldine form), and the pair of redox peaks C2/A2 is associated with the faradaic transformation of emeraldine-pernigraniline^38^. Evidently, in comparison with that of PANI/RGO, RGO/PANI/RGO paper electrode exhibits larger CV area, indicating a higher specific capacitance of RGO/PANI/RGO paper^39^.

Fig. 4b shows the galvanostatic (GV) charge/discharge curves of the as-prepared RGO/PANI/RGO paper electrode at different current densities. During the charging and discharging steps, the charge curve of RGO/PANI/RGO paper is almost symmetric to its corresponding discharge counterpart with a slight curvature, which is similar to that of PANI/RGO paper. However, the “IR drop” of RGO/PANI/RGO paper electrode is much lower than that of PANI/RGO paper electrode, as shown in Fig. 4c, which reflects the lower internal resistance of RGO/PANI/RGO paper electrode with respect to that of PANI/RGO paper electrode. As a matter of fact, the PANI-based electrode material usually has large internal resistance as it is fully charged or discharged because of its poor electrical conductivity. However, in this work, the electrical conductivity of PANI/RGO paper electrode has been dramatically increased by further coating of an ultrathin RGO film on the PANI/RGO paper to form sandwich-structured RGO/PANI/RGO paper, which enhances the electrical conductivity by the large-scale π–π conjugation between PANI and RGO, and thus facilitates the charge transfer, resulting in a lower internal resistance and less wasted energy. The specific capacitances (C_s_) of RGO, PANI/RGO and RGO/PANI/RGO paper electrodes calculated from their charg/discharge curves are illustrated in Fig. 4d. At a current density of 1 A g^−1^, the C_s_ of RGO, PANI/RGO and RGO/PANI/RGO paper are 55 F g^−1^, 522 F g^−1^ and 581 F g^−1^, respectively. RGO/PANI/RGO paper not only exhibits the highest specific capacitance values but also maintains them well at high current density compared to other electrodes. Its specific capacitance is preserved 64.4% (from 581 to 374 F g^−1^) as the current density increases from 1 to 10 A g^−1^, which is superior to many other graphene nanohybrid electrodes such as graphene/CNT^40^, graphene/MnO_2_^41^,^42^ and graphene/PANI^43^,^44^. More importantly, we have shown that the power densities of RGO/PANI/RGO paper are also higher than those of PANI/RGO paper at a variety of current density of 1, 2, 5, 8 and 10 A g^−1^ (Fig. 4e). Therefore, it can be ensured that increasing the electrode thickness by extra RGO does not scarify the power density of RGO/PANI/RGO paper electrode.

The significant improved supercapacitive performances of RGO/PANI/RGO paper electrode in terms of high specific capacitance and good rate capability can be recognized as: i) RGO paper possesses highly electrical conductivity and mechanical strength, which acts as an ideal current collector for high-performance supercapacitor; ii) After in situ electropolymerization of PANI nanofibers on ROG paper, the PANI/RGO exhibits a better electrochemical capacitive characteristic and superior reversible redox reaction due to the incremental pseudocapacitive contribution the overall capacitance. iii) Although PANI exhibits poor electrical conductivity, this drawback can be overcome by further coating an ultrathin RGO layer on it. The strong π–π interactions between the highly conductive RGO layer and the PANI chains in the RGO/PANI/RGO could provide a good conducting network, which facilitates the redox reaction of PANI component and increases the pseudocapacitance of the nanohybrid paper electrode.

The Nyquist plots of RGO, PANI/RGO and RGO/PANI/RGO paper electrodes have been demonstrated in Fig. 4f with an expanded view of the high-frequency region and an equivalent circuit in the inset. All plots feature the most vertical line in a low-frequency region, indicating a nearly ideal capacitive behavior. The x-intercept of the Nyquist plots represents the equivalent series resistance (ESR) for supercapacitor electrodes and the charge transport resistance. The charge transfer resistance of PANI/RGO paper (23.8 Ω) is larger than that of RGO paper (9.7 Ω) because of the lower conductivity of PANI. When PANI/RGO paper has been covered by a layer of highly conductive RGO, the ESR of the sandwich-structured RGO/PANI/RGO paper reduces to 12.7 Ω, reflecting the higher conductivity achieved in RGO/PANI/RGO system. These are consisted with the results obtained by GV charge–discharge measurements.

**Figure 5:**
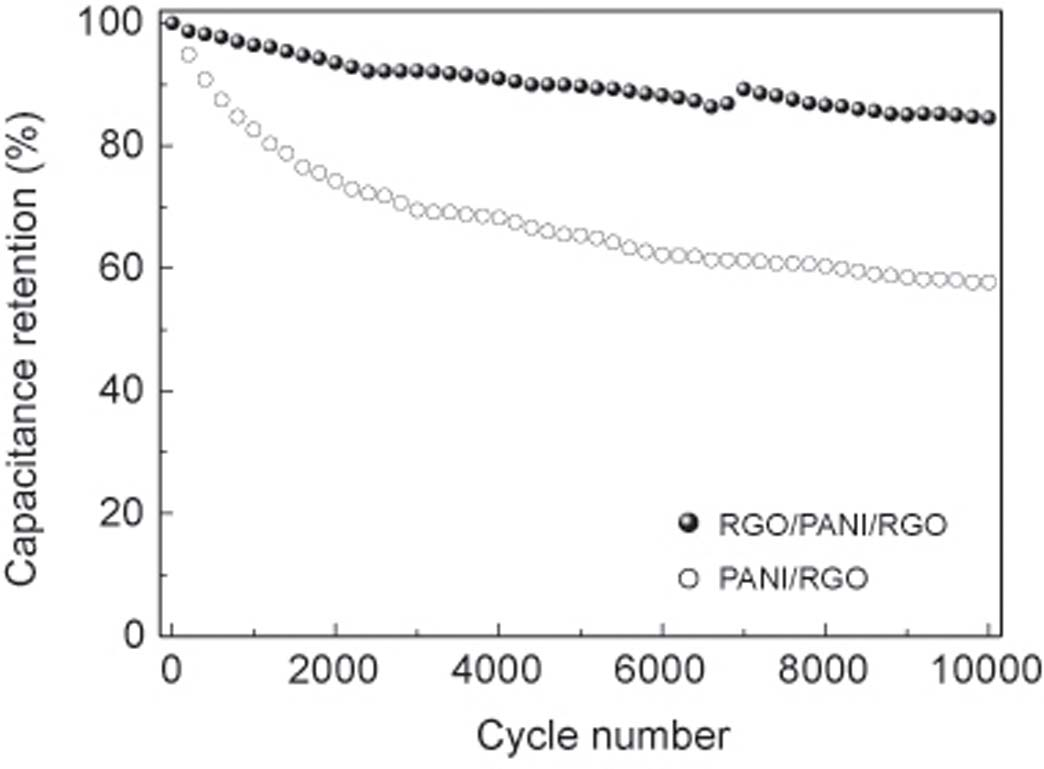
Cycle performance of RGO/PANI/RGO and PANI/RGO paper electrodes at 10 A g^−1^ over 10000 cycles.

Fig. 5 shows the cycling performance of RGO/PANI/RGO paper with controlled PANI/RGO paper. The RGO/PANI/RGO paper retains 85% of its initial capacitance after 10000 cycles, which outperforms that of PANI/RGO paper (57%). As active electrode materials for supercapacitors, the conducting polymers often suffer from poor cyclic stability because of the cyclic mechanical stress caused by material swelling and shrinking. However, the coating of mechanically stronger graphene nanosheet on the surface of network PANI nanofibesr can enhance the restraint of shrinkage and swelling of PANI chains during numerous charge/discharge cycles. In addition, the graphene layer on PANI/RGO paper can also mechanically stabilize the PANI nanomaterials and keep them bound together during the cycling tests to avoid PANI material loss from electrode surfaces, which effectively improve the cycling performance of the resultant nanohybrid electrode.

**Figure 6:**
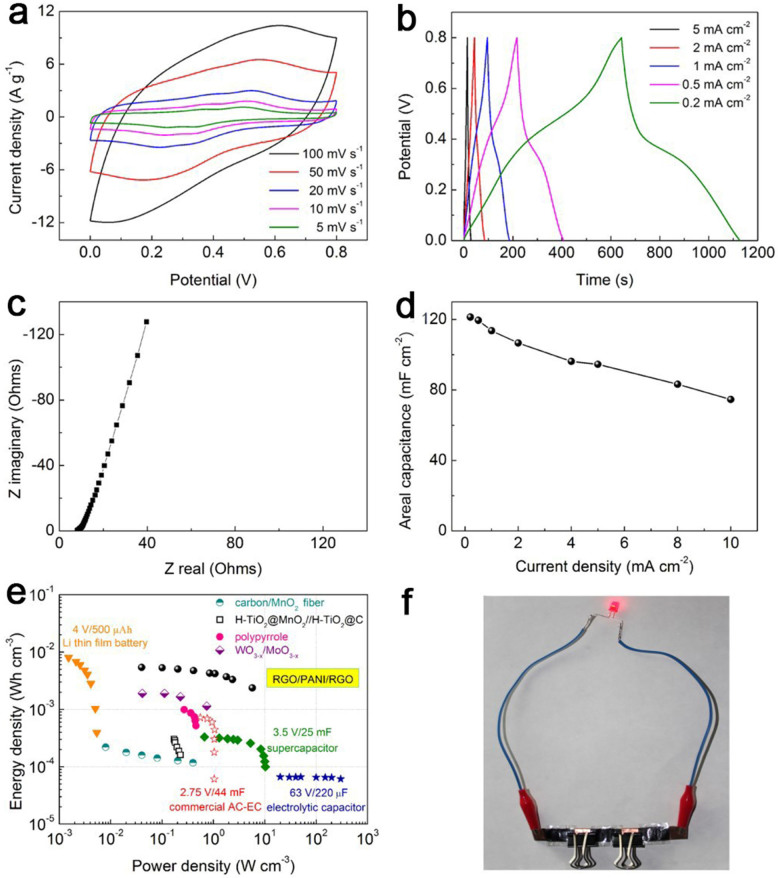
(a) CV curves, (b) GV charge–discharge curves, (c) Nyquist plot and (d) areal capacitance versus discharge current for the solid-state device. (e) Ragone plot of energy density versus power density for different solid-state device. (f) Photograph of a red LED lit by in-series solid-state SCs with three units.

To make flexible SCs entirely wearable, the electrolyte needs to be fully encapsulated in the devices without any leakage. Taking this into account, the use of solid gel electrolytes is more suitable than liquid electrolytes of H_2_SO_4_ for further applications of flexible SCs^45^. CV curves of the solid-state device show a rectangular shape and two pairs of redox peaks at different scan rates from 5 to 100 mV s^−1^ (Fig. 6a), indicating a pseudocapacitive behavior. Moreover, the symmetrical GV charge/discharge curves reveal the good capacitive performance as well (Fig. 6b). The Nyquist plots of the solid-state SC device demonstrated in Fig. 6c feature almost vertical line in a low-frequency region, indicating a nearly ideal capacitive behavior. Furthermore, the charge-transfer resistance (*R*_ct_) of the solid-state SC is about 4.5 Ω, which reveals the low resistance of the redox reaction of the solid-state SC device. The as-prepared device maintains its 88% areal capacitance (107 mF cm^−2^) as discharge current density increases from 0.2 to 2 mA cm^−2^ (Fig. 6d). Even when the current density increases to 10 mA cm^−2^, it still maintains 62% areal capacitance.

The Ragone plots for the electrode materials are shown in Fig. 6e. For the electrode materials among the current density measurement range, it has a volumetric energy density of 5.4 mWh cm^−3^ (10.79 wh kg^−1^), which outperforms many previously reported solid-state SCs, such as polypyrrole-coated paper symmetric SC (1 mWh cm^−3^)^10^, carbon/MnO_2_ fiber symmetric SC (0.22 mWh cm^−3^)^30^, H-TiO_2_@MnO_2_//H-TiO_2_@C asymmetric SC (0.3 mWh cm^−3^)^31^, and WO_3-x_/MoO_3-x_ asymmetric SC (1.9 mWh cm^−3^)^32^. Three solid-state SC (10 mm × 25 mm) units connected in series can light a red light-emitting diode (LED) at 2.4 V, as shown in Fig. 6f.

In summary, we reported a facile and scalable method to prepare freestanding sandwich-structured graphene/PANI/graphene nanocomposite paper and demonstrated its practical application in flexible all-solid-state SCs. The graphene/PANI/graphene nanohybrid paper exhibited three unique features: i) the constituent graphene paper fabricated by the simple and scalable printing method and subsequent HI treatment possessed high conductivity, light weight, mechanical robustness and chemical stability, which functions itself as an ideal current collector for SC architecture as well as the substrate for the further loading of active electrode materials on it. ii) PANI nanofibers synthesized by a green in situ electropolymerization method have effectively improved the electrochemical performance of the nanohybrid paper. iii) dip-coating of an ultrathin graphene layer on the surface of the porous PANI nanofibers has endowed the sandwich-structured graphene/PANI/graphene nanohybrid paper electrode with enhanced specific capacitance and increased cycling stability. The resultant flexible symmetric SC based on the graphene/PANI/graphene nanohybrid paper and polymer gel electrolyte exhibits high energy density, high power density and excellent cycling stability, which makes it promising for the future applications in flexible energy-related device and modern wearable/portable electronics. It is worth noting that this modular approach for the fabrication of freestanding sandwich-structured graphene nanohybrid paper is not just restricted to the graphene/PANI/graphene system, but also contributes to the design of hybrid nanoarchitectures with tailored compositions and functions, which provides new insights on designing high-performance paper electrode for flexible energy-related device and modern wearable/portable electronics.

## Methods

### Fabrication of RGO/PANI/RGO Composite Paper

First, well dispersed GO solution (5mg mL^−1^) was dropped onto a piece of commercial printing paper surface. Then, the rod was rolled back and forth to make the GO solution covered uniformly on the paper. After subsequent air-drying, a uniform GO paper are obtained. The GO paper was reduced by means of soaking in the HI solution (45 wt%) for 10 minutes at room temperature, during this process, bubbles generated from the cellulose papers prompt the thin film peeling off the paper in a self-releasing way. Subsequently, the RGO paper was rinsed with a saturated sodium bicarbonate solution, water and methanol and dried by vacuum drying at 80°C. RGO paper was then coated by a layer of uniform porous nanofiber network of PANI via in situ electropolymerization. In the typical procedure, the PANI nanofibers were synthesized by a two-step method on a conventional three-electrode system in a 1 M HCl electrolyte containing 0.3 M aniline monomer. The first step of the nucleation of PANI was performed at a constant potential of 0.8 V for 1 min at room temperature. In the second step, the nanowires were grown under a constant current condition with current density of 2 mA cm^−2^ 46. After that, the PANI layer was covered with an ultrathin GO film through dip-coating method. The resultant GO/PANI/RGO paper was also soaked in the HI solution for a few minutes at room temperature, and the RGO/PANI/RGO paper was obtained.

### Characterization

Scanning electron microscopy (SEM) images were taken on a FESEM instrument (SIRION 200, FEI, Nederland). X-ray photoelectron spectroscopy (XPS) measurements were performed on VG ESCALAB 250 spectrometer with monochromatic Al Kα (1486.71 eV) X-ray radiation (15 kV and 10 mA) and hemispherical electron energy analyzer. Xray powder diffraction (XRD) patterns were recorded using a diffractometer (X' Pert PRO, Panalytical B.V., Netherlands) equipped with a Cu Kα radiation source (λ = 1.5406 Å). Fourier transform infrared (FTIR) spectra were obtained on Bruker VERTEX 70 FTIR spectrophotometer (Germany).

### Electrochemical Tests

The electrochemical characteristics were evaluated by cyclic voltammetry, galvanostatic charge/discharge and electrochemical impedance spectroscopy using a CHI 660E electrochemical workstation (Shanghai CH Instruments Co., China). A conventional three-electrode system was adopted. The working electrode was RGO/PANI/RGO paper, and the counter and reference electrodes were platinum plate and saturated calomel electrode (SCE), respectively. The aqueous electrolyte is 1 M H_2_SO_4_. In the case of all-solid-state SCs, two piece of RGO/PANI/RGO paper were immersed into a hot clear solution of PVA/H_2_SO_4_ electrolyte for 5 minutes, then taken out and assembled together with a piece of filter paper sandwiched in between the two electrodes. Finally, the device was solidified for 4 h at room temperature. The weight being used in the calculation of capacitance is referring to the weight of the entire device. The gel electrolyte was fabricated by mixing 6 g H_2_SO_4_ and 6 g PVA in 60 mL deionized water and heated up to 85°C for 1 h under vigorous stirring. CV measurements were conducted in a voltage range of 0–0.8 V with voltage sweep rates from 5 to 100 mV s^−1^. Galvanostatic charge/discharge measurements were carried out at different current density from 1 to 10 A g^−1^. EIS tests were performed from 10 mHz to 100 kHz with an amplitude of 5 mV referring to open circuit potential^47^.

## Supplementary Material

Supplementary Information Supporting Information
