# Supplementary material for: Scalable Synthesis of Freestanding Sandwich-structured Graphene/Polyaniline/Graphene Nanocomposite Paper for Flexible All-Solid-State Supercapacitor
Source: Sci Rep. 2015 Mar 23;5:9359. doi: 10.1038/srep09359 (PMC4369721; doi:10.1038/srep09359)
Supplement: Supplementary Information — Supporting Information [file srep09359-s1.docx]

Supporting Information

**Scalable Synthesis of Freestanding Sandwich-structured Graphene/Polyaniline/ Graphene Nanocomposite Paper for Flexible All-Solid-State Supercapacitor**

Fei Xiao, Shengxiong Yang, Zheye Zhang, Hongfang Liu, Junwu Xiao, Lian Wan, Jun Luo, Shuai Wang* & Yunqi Liu


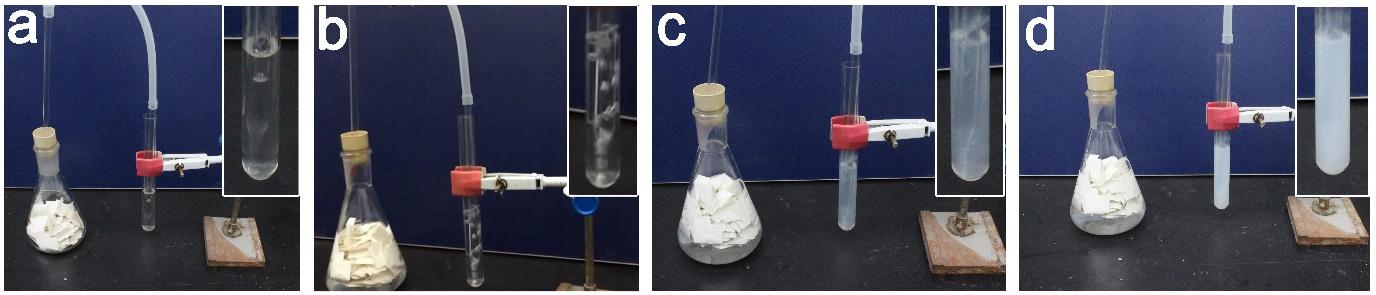


**Figure S1**. Photographs of experiment that A4 paper reacted with 1 M HCl solution. (a) Before reaction, lime water was clear. (b) After added 1 M HCl solution into the conical flask, a lot of bubbles emerged immediately. (c-d) As the reaction proceeded, lime water became turbid, which turned out that the gas produced during the reaction is carbon dioxide.


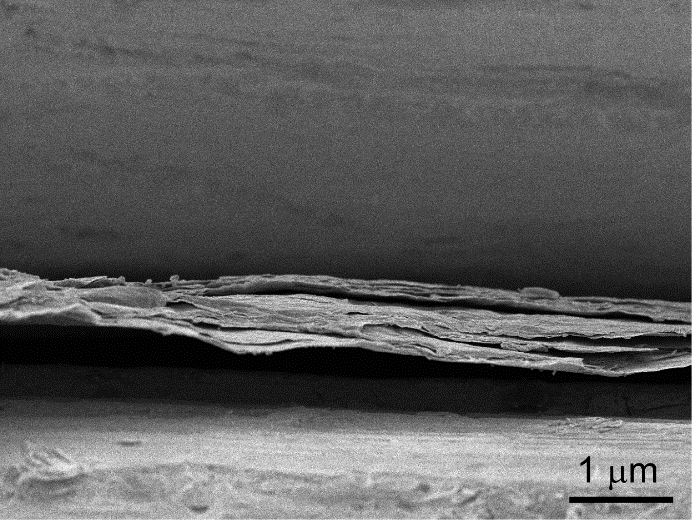


**Figure S2**. SEM image of ultrathin RGO paper with a thickness of about 800 nm, prepared by the proposed printing technique and bubbling delamination method.


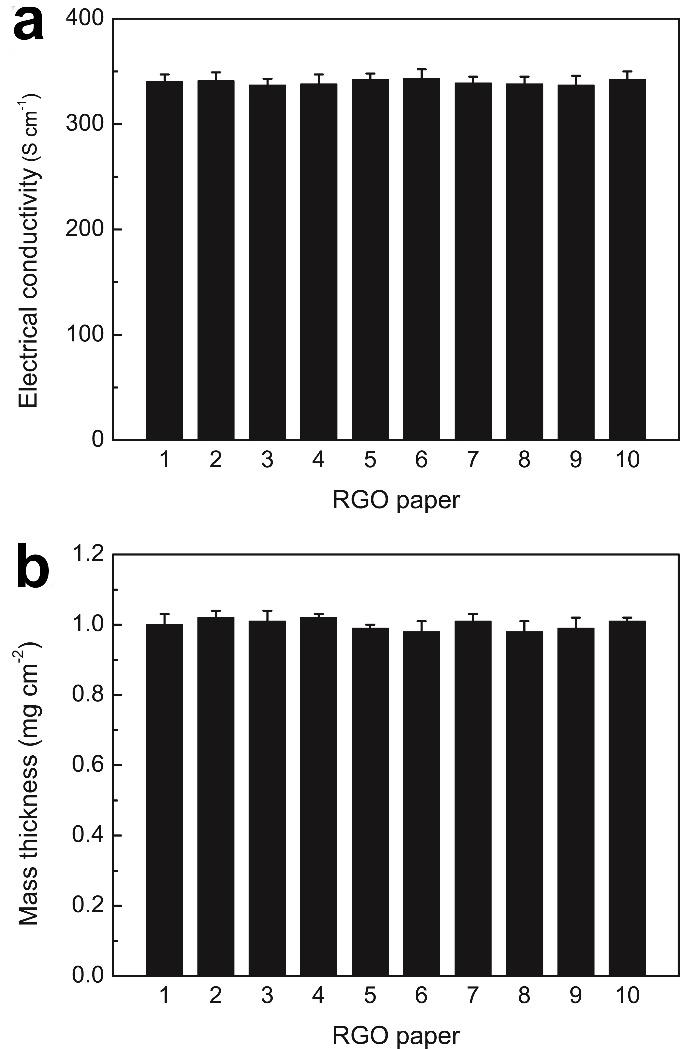


**Figure S3**. (a) The electrical conductivity and (b) mass thickness of ten different RGO papers.
